# Supplementary material for: Complexome profiling of the Chlamydomonas psb28 mutant reveals TEF5 as an early PSII assembly factor
Source: Plant Cell. 2025 Mar 17;37(6):koaf055. doi: 10.1093/plcell/koaf055 (PMC12164590; doi:10.1093/plcell/koaf055)
Supplement: koaf055_Supplementary_Data [file koaf055_supplementary_data.zip › psb28_interactive.zip!/plots/intensity/qualityPlots/peptides/TOC34_Cre06.g252200.t1.2.html]

### Cre06.g252200.t1.2

Trivial name: TOC34  
SeqCoverage [median]: 13.60  
Mass: 44.54 kDa  
  
 

| Sequence | MissedCleavage | Mass [Da] | wtIntensity | muIntensity | Score |
| --- | --- | --- | --- | --- | --- |
| AIIDAISSTFGR | 0 | 1249.67 | 282.18 | 0.00 | 107.79 |
| IAEDVR | 0 | 701.37 | 738.92 | 0.00 | 91.58 |
| KQLTVLLLGK | 1 | 1111.73 | 0.00 | 804.62 | 42.72 |
| LQADTDITTTVVR | 0 | 1431.76 | 0.00 | 1359.06 | 71.56 |
| QLTVLLLGK | 0 | 983.64 | 334.83 | 0.00 | 53.17 |
| QVAVGNSEVDGFR | 0 | 1376.67 | 0.00 | 1205.53 | 90.05 |
|  |  |  | 1355.9 | 3369.2 |  |
